# The Clinical Relevance of Overnight Oximetry in the Diagnosis of Intermittent Desaturations and the Need for Home Oxygen in the Near-Term and Term Infant

**DOI:** 10.3390/children12101341

**Published:** 2025-10-05

**Authors:** Amelia N. Noone, Chad C. Andersen, Tara M. Crawford, Michael J. Stark

**Affiliations:** 1 The Robinson Research Institute, The University of Adelaide, Adelaide 5006, Australia; amelia.noone@adelaide.edu.au (A.N.N.);; 2 The Department of Neonatal Medicine, The Women’s and Children’s Hospital, Adelaide 5006, Australia

**Keywords:** desaturations, intermittent hypoxia, infant, overnight oximetry, near-infrared spectroscopy, cerebral tissue oxygen saturation

## Abstract

While intermittent desaturations are a common occurrence in near-term and term infants, these may not be benign events with fluctuations in oxygen saturation associated with later neurodevelopmental impairment. Further, intermittent desaturation events do not necessarily result in intermittent hypoxia (IH). Polysomnography is the gold standard to diagnose intermittent desaturations in infants; however, it remains an expensive, inaccessible test. Therefore, overnight oximetry; an economical and more readily available test, is routinely used in this population. Overnight oximetry employs measurement of peripheral cutaneous arterial oxygen saturation (SpO_2_) alone to inform clinical management of intermittent desaturations. Management strategies include discharging near-term and term infants on low-flow long-term oxygen therapy (LTOT), typically for upwards of six months. Oxygen saturation targets for neonates have been widely studied. However, interpretation of overnight oximetry is problematic due to a lack of established reference ranges with current approaches still based on limited evidence. This raises questions of the clinical relevancy of overnight oximetry in infants for diagnosing IH and the resultant need for LTOT. Given the association between IH and later neurodevelopmental impairment, concurrent measurement of cerebral tissue oxygen saturation by near-infrared spectroscopy (NIRS) may better identify those near-term and term infants in need of LTOT. Here we review the emerging evidence for the clinical use of cerebral NIRS and the relevance of overnight oximetry in identifying IH in near-term and term newborns and its potential role in identifying those requiring LTOT.

## 1. Introduction

Maintenance of normal oxygen saturations in the newborn period is a cornerstone of neonatal clinical practice. However, desaturation episodes, which are common problem in near-term and term infants, do not necessarily result in intermittent hypoxia (IH), characterised in the literature by short, repetitive cycles of fluctuations in oxygenation [1]. In this review, IH refers to SpO_2_-defined IH, reflecting recurrent desaturation events measured by pulse oximetry. We acknowledge that SpO_2_ is an indirect marker and thus, may not capture all aspects of tissue-level hypoxia; however, it remains the most practical measure in neonatal studies. While early postnatal IH may not always correlate with adverse long-term outcomes, IH in the neonatal period is reported to be associated with later neurodevelopmental impairment [2]. The reasons for this variable effect remain unknown. This has resulted in increasing use of long-term oxygen therapy (LTOT) in this population, defined as the ongoing requirement for oxygen to manage chronic hypoxemia following discharge home [3,4]. However, there remains significant practice variation with respect to both initiation and subsequent weaning from LTOT [5]. Whilst the most common indication for LTOT is chronic lung disease related to bronchopulmonary dysplasia in newborns born between 23 and 31 weeks’ gestation, near-term or term-born newborns without a major congenital abnormality make up 19% of newborns discharged home on LTOT [6]. In this population, conditions that result in the need for LTOT include meconium aspiration syndrome with pulmonary hypertension, structural lung disease, and sleep-disordered breathing [3].

The gold standard for IH diagnosis and the need for LTOT is overnight polysomnography. This is a resource- intensive, expensive, and frequently inaccessible test. As a result, most clinical decisions to send an infant home on oxygen are based solely on the less refined but more readily available overnight oximetry, despite its inherent limitations. Overnight assessment is preferred as sleep is characterised by changes in muscle tone and control, responsiveness to apnoea, and stability of respiratory control, with falls in minute ventilation and functional residual capacity [7]. This is, however, problematic due to the absence of standard neonatal reference ranges for continuous overnight oximetry, and the lack of diagnostic criteria defining its relationship to clinically relevant IH in this patient group [1]. As a result, clinical interpretations of overnight oximetry may vary and consequently affect management strategies, including LTOT use.

The impact of immature ventilatory control, contributing to hypoxemia, continues to be the emphasis for ongoing large-scale trials focusing on very preterm newborns [8]. However, determining whether abnormal SpO_2_ used to define IH in the near-term and term infant, is a clinical problem with real short- and long-term consequence remaining as a knowledge gap. Here we review current clinical practices for near-term and term infants with unexplained oxygen requirements. Furthermore, we discuss the relevancy of overnight oximetry to diagnose IH and guide the use of LTOT in near-term and term newborns. Finally, we address potential trial designs utilising near-infrared spectroscopy (NIRS) to further investigate the clinical relevance of overnight oximetry. A specific focus of this narrative review is the use of additional, adjunctive monitoring to establish if overnight oximetry accurately reflects episodes of cerebral tissue hypoxia in near-term and term infants with IH.

## 2. Methods of the Review

A literature search was conducted on PubMed, Scopus, Embase, Cochrane, CINAHL, and Science Direct to identify relevant studies for the current narrative review. Key search terms included: term, preterm, infant, neonate, newborn, overnight oximetry, pulse oximetry, nocturnal oximetry, intermittent hypoxia, sleep-disordered breathing, neurodevelopmental outcomes, oxygen therapy, home oxygen, long-term oxygen therapy, oxygen use, caffeine therapy, near-infrared spectroscopy, cerebral oxygen saturation, and cerebral saturation. Studies published in English between 1978 and 2025 were included, and animal studies were excluded. While the narrative review focused on summarising trends, clinical implications, and design considerations for future research, no formal critical appraisal tool was applied, and we cannot exclude the possibility that some relevant studies may have been missed.

## 3. Defining Intermittent Hypoxia

IH episodes are frequently experienced by preterm newborns < 31 weeks’ gestation during early postnatal life [2,4]. In this population, the frequency of IH events, as indicated by SpO_2_, steadily increases from <50 events per day in the first week of life, reaching a plateau of 100–200 events by the end of the first postnatal month [9,10], with the duration of the IH events shortening but the degree of hypoxia increasing [11]. The primary triggers of these IH events are immature respiratory control, resulting in apnoea and respiratory pauses, and obstructed inspiratory efforts, superimposed on a developmentally immature lung with low basal functional residual capacity due to atelectasis and loss of lung volume [12].

Episodes of IH as indicated by SpO_2_ are also commonplace in near-term and term newborns [13]. Even in this more mature newborn population, the pattern of breathing can be inherently unstable during sleep [14]. Central apnoea, characterised by a brief arrest in ventilation due to reduced respiratory drive within the central nervous system, is common in infants. It often results from immature ventilatory control and typically manifests as periodic breathing in the postnatal period. These episodes occur predominantly during episodes of rapid eye movement (REM) sleep [15] and result in transient falls in oxygen saturation [16]. Desaturations, occurring when SpO_2_ drops below a defined threshold, are more frequent in near-term and term infants during active sleep [17]—the predominant sleep state during early postnatal life [18]—compared with quiet sleep [17,18,19]. This may be due to hypoventilation, low pulmonary oxygen reserve, and increased oxygen consumption [20,21], which are more likely to occur in active sleep [22]. Importantly, the proportion of active sleep decreases across the first months after birth. Although near-term infants experience a higher frequency of IH, as indicated by SpO_2_, than term infants, both shortly after birth and at term-equivalent age, by 45 weeks postmenstrual age their IH frequency declines to that of term-born infants of the same postnatal age [22,23].

## 4. Diagnosis of Intermittent Hypoxia in Near-Term to Term Infants

Polysomnography is the gold standard for diagnosing IH in infancy and childhood. This is a multi-channel test with continuous measurement of central, occipital, and frontal electroencephalogram (EEG), electro-oculograms (EOG), electromyogram (EMG), electrocardiogram (ECG), nasal pressure, pulse oximetry, end-tidal CO_2_, and thoracic and abdominal respiratory inductance plethysmography. However, access to paediatric sleep units to conduct polysomnography can be limited. Coupled with inaccessibility, polysomnography is expensive and is often subject to long wait lists. This has resulted in a model of care where most newborns referred for formal paediatric respiratory assessment of an unexplained oxygen requirement undergo overnight oximetry and the subsequent commencement of LTOT, prior to later polysomnography.

At the bedside, oximetry provides continuous and non-invasive monitoring of peripheral saturation during the newborn period. At a practical level, pulse oximetry only offers a localised assessment of oxygen saturation in a specific pulsatile vessel [24]. SpO_2_ is estimated by calculating the percentage of oxygenated haemoglobin in arterial blood [25]. As such, pulse oximetry has its limitations, and is sensitive to movement, poor peripheral perfusion, dark skin pigmentation, and light [26,27]. It does not capture oxygen saturation within the venous compartment and cannot be used to estimate end-organ oxygenation [28]. As an upstream parameter, oxygen saturation does not directly represent metabolic oxygen use at the tissue level. Importantly, there is also an inherent delay in pulse oximetry in signalling the actual clinical event of changes in oxygen saturation [29], a drawback in its use to investigate the impact of IH in the newborn.

Overnight oximetry metrics are not fully standardised for neonates, and definitions can vary depending on the device and software used. For clinical reporting in neonates, typical indices include the number of desaturation events (either ≥3% or ≥4% drop in SpO_2_, although there is no accepted definition of event duration) [7], cumulative time spent with oxygen saturation < 90% and <80%, as well as the highest, lowest, and mean values (with standard deviations) for both heart rate and SpO_2_ [13]. However, the specific indices, their calculation, and how the data are displayed can vary depending on the software used to generate the report [7]. In research settings, additional indices may include the depth and duration or area under the curve for desaturation. Technical variabilities between overnight oximetry device settings can make comparison and interpretation problematic [30]. Averaging time is an important parameter of overnight oximetry, as longer averaging times (8–16 s) are beneficial in reducing motion artefacts, whereas shorter averaging times (2–3 s) are more sensitive to fluctuations in oxygen saturation that can occur rapidly. In a paediatric cohort, it is recommended to use a shorter averaging time; however, it is important to note that these definitions are not directly applicable to the neonatal population [7]. The Thoracic Society of Australia and New Zealand recommends that infants with chronic neonatal lung disease maintain overnight oximetry targets between 93 and 95%, with less than 5% of total recording time below 90% SpO_2_ [31]. This position aligns with the European Respiratory Society and American Thoracic Society guidelines, although the supporting evidence remains limited [31].

The most significant limitation of pulse oximetry is the relatively limited data on which neonatal reference ranges are based (Table 1). For the preterm newborn, oximetry is routinely employed throughout the postnatal period to maintain targeted oxygen saturation ranges, thereby avoiding both inadequate and excessive oxygen exposure and related newborn morbidity and mortality [32]. Contemporary clinical practice is based on the findings of the Neonatal Oxygenation Prospective Meta-Analysis (NeOProM), which included five randomised, double-blinded, multicentre trials (SUPPORT, COT, BOOST-NZ-2006–2012, BOOST-II UK 2007–2014, and BOOST II AUS 2006–2013) of infants < 28 weeks’ gestation randomised to a lower (85–89%) or higher (91–95%) SpO_2_ target range [33].

Evidence on SpO_2_ patterns in near-term to term infants remains limited. However, earlier studies, notably those by Poets et al., remain an important reference until more contemporary and comprehensive data are available [1]. For the well near-term and term newborn not requiring supplemental oxygen, only minor developmental changes in arterial oxygen saturation are observed over the first four weeks of life [1]. In the first 48 h of life, the mean SpO_2_ for this group of newborns is >95% [34]. Time with SpO_2_ > 90% is significantly lower in near-term newborns compared to those born at term, with 7% of near-term and term newborns weighing < 2.5 kg spending up to 7% of the time with SpO_2_ ≤ 90% [34]. SpO_2_ then remains relatively stable across the first postnatal month (median baseline SpO_2_ 97.6% during week 1 versus 98.0% during week 4) [1,35]. Beyond the immediate postnatal period, the Collaborative Home Infant Monitoring Evaluation (CHIME) study reported baseline SpO_2_ levels in healthy term infants at 2 to 25 weeks of age of >95%, but frequent acute IH events (SpO_2_ < 90%) during the early postnatal weeks were also common [36]. A follow-up study comparing 103 preterm infants born at <1750 g and ≤34 weeks postmenstrual age with 99 healthy term infants found median baseline SpO_2_ was approximately 98% for both gestational age groups. IH episodes, defined as an acute decrease in SpO_2_ of at least 10% from the preceding stable baseline sustained below 90% for ≥5 s, were observed in 74% of preterm and 62% of the term infants [35]. Over time, the incidence of IH episodes and the number of seconds per hour below 90% during IH in the preterm group fell to be no different to that of the term group by 43 weeks PMA [35].

Despite this observational data, there remains no consensus definition of IH or desaturation event with respect to SpO_2_ or duration of the event in the newborn infant [23]. In the context of clinical trials, the most common definition of IH is a decrease in oxygen saturation to <80% [37]. However, definitions based on expert opinion do exist for older infants and children. The Australasian Sleep Association (ASA) paediatric guidelines for evaluating paediatric obstructive sleep apnoea (OSA) define a desaturation as a ≥3% fall in peripheral cutaneous oxygen saturation from baseline [7]. Additional definitions employed include clusters of desaturations (≥5 desaturations within a 10–30 min period of sleep) and the incidence of falls in SpO_2_ below 90%, 85%, and 80% [7]. It is important to note the ASA guideline is only validated in a paediatric cohort > 12 months of age and is specific for patients with suspected OSA.

In 2012, the American Academy of Sleep Medicine guidelines lowered the desaturation threshold for diagnosing OSA and central sleep apnoea (CSA) in older children from 4% to 3% [38]. For either definition of IH [7], little is known about the frequency of these episodes in young infants. Evans and colleagues were the first to report both 4% and 3% desaturation indices in healthy term newborns undergoing overnight pulse oximetry at home at 1 and 3–4 months of age [15]. In this study, 45 babies were studied at 1 month, 38 babies at 3–4 months, and paired studies were available for comparison for 32 [15]. Both the 4% and 3% indices were greater than in older children, with each falling significantly by the 3 month overnight oximetry [15]. Whether either the ASA or American Academy of Sleep Medicine guidelines and definitions are appropriate for newborns in the postnatal period is unknown. Given practice variation, specifically the importance placed of overnight oximetry-defined IH in the initiation of LTOT in this population, specific, evidence-based neonatal guidelines are clearly necessary.

## 5. Intermittent Hypoxia and Impaired Neurodevelopment in Near-Term to Term Infants

In the near-term and term neonatal population, data on the morbidity associated with IH is limited. Further, confounding morbidities and factors impact the ability to ascertain the direct impact of IH on longer-term outcomes. Such confounders may include illness severity, underlying genetic predisposition, and socioeconomic circumstances that shape both health and developmental opportunities. The most robust evidence exists for the most preterm newborns with the greatest susceptibility to frequent IH. Clinical evidence suggests associations between delayed resolution [39,40], increased frequency or severity of apnoea/bradycardia episodes, and neurodevelopmental impairment [41,42] (Table 2).The multicentre Canadian Oxygen Trial investigated 1019 preterm infants and found that during the first 2–3 months of age, hypoxemic events (SpO_2_ < 80%) ≥ 1 min were associated with adverse outcomes at 18 months [43]. This included language and/or cognitive delay, reflected by a score of 1 standard deviation below the mean (<85) on the Bayley Scales of Infant and Toddler Development (BSID), 3rd Edition [43]. The relative risk for the primary outcome of late death or disability was 1.66 (95% CI, 1.35–2.05) for long hypoxemic events (≥1 min) and 1.01 (95% CI, 0.77–1.32) for short (<1 min) episodes [43]. The data was also analysed following conversion of percentage time spent hypoxemic into deciles, with infants in the highest decile for time spent < 80% SpO_2_ being three times more likely to develop cognitive or motor delay than those in the lowest decile.

The CHIME study group investigated cardiorespiratory events detected by home monitoring and its effects on neurodevelopmental outcome at one year of age in 256 infants in two gestational age groups, preterm (<1750 g and 34 weeks) and term [44]. Cardiorespiratory events were defined as apnoea ≥ 20 s, or heart rate < 60 beats per minute (bpm) for ≥5 s or <80 bpm for ≥15 s if <44 weeks post-conceptional age, or <50 bpm for ≥5 s or <60 bpm for ≥15 s if >44 weeks post-conceptional age. In both groups, adjusted Mental Developmental Index (MDI) scores assessed using the BSID 2nd Edition in infants with >5 cardiorespiratory events compared to those with no events were 4.9 points lower for the preterm group and 5.6 points lower for the term group [44]. A limitation of this study is that 44% of eligible infants did not return for BSID testing, and those who did shared favourable demographic factors, limiting the generalisability of the findings [44]. Additionally, it is important to acknowledge that this data provides no insight into whether the lower MDI was a direct consequence of the cardiorespiratory events or whether both had a common underlying cause. Further, the exposure analysed was not IH, rather a combination of prolonged apnoea and/or bradycardia events. Finally, it remains unknown whether cumulative time, severity, or frequency of these events, or most likely a combination of all three, is the primary driver of potential adverse neurodevelopmental outcomes.

There is also evidence for possible associations between specific pathological causes for IH and adverse neurodevelopmental outcomes. Sleep-disordered breathing, because of upper airway dysfunction, can increase respiratory effort and alterations in respiratory patterns leading to IH in the neonatal period. Snoring, the mildest form of sleep-disordered breathing, at any age is not a benign condition. Piteo and colleagues assessed 16 infants with parental-reported snoring, and 88 non-snoring healthy controls, with infants who snored frequently (≥3 nights/week) scoring lower for cognitive development on the BSID 3rd Edition [45]. Despite preliminary analyses showing no correlation between either variable and developmental scores, infants who snored frequently tended to be male, were breastfed for fewer days, and with a higher intake of formula [45]. Respiratory arousal index has also been shown to be significantly correlated with MDI. Montgomery-Downs and Gozal reported neurodevelopmental outcomes at 8–9 months of age in a cohort of 35 healthy infants (8.2 ± 0.4 months) who underwent polysomnography [46]. Arousals related to snoring, rather than spontaneous arousals or those linked to CSA with desaturations (≥4%), were associated with lower MDI scores and were found to be exacerbated by exposure to a smoking environment at home [46].

Similarly, disorders that alter the structure of the upper airway also result in sleep-disordered breathing and increase the likelihood of IH. Smith and colleagues reported polysomnography-diagnosed measures of sleep-disordered breathing (performed at 2.7 ± 2.1 months) prior to surgical intervention, and subsequent neurocognitive (BSID 3rd Edition), quality of life, and growth assessment at 3 years [47]. Although no association was seen between oxygen desaturation index (number of oxygen desaturation events ≥ 3% during sleep divided by the total sleep time) and later outcomes, a greater obstructive-mixed apnoea–hypopnea index corresponded with lower global behaviour Infant/Toddler Quality of Life Questionnaire score, and a smaller proportion of REM sleep was associated with reduced cognitive performance. Despite the available evidence, many of these cohorts include heterogeneous exposures, including apnoea, bradycardia, and other co-morbid pathologies. As such, the extent to which isolated, brief desaturations detected by SpO_2_ in otherwise healthy term infants contribute to long-term outcomes remains uncertain. Further confounders including illness severity, genetic vulnerability, or socioeconomic conditions may influence trajectories of health and development. Further research that accounts for these potential confounders is required to clarify the impact of IH on long-term neurodevelopmental outcomes in near-term and term newborns.

## 6. Current Approaches to the Management of IH in the Near-Term to Term Newborn

### 6.1. Home Oxygen Therapy

Oxygen is a widely used therapy in neonatology, and home oxygen can facilitate earlier discharge. For the purpose of this review, and in line with the British Thoracic Society, LTOT is defined as a continuous requirement of oxygen at home following primary hospital discharge [4]. Critically, as outlined in the American Thoracic Society Home Oxygen Therapy for Children Clinical Guideline “although home oxygen therapy is commonly required in the care of children, there is a striking lack of empirical evidence regarding implementation, monitoring, and discontinuation of supplemental oxygen therapy” [48]. This guideline recommends LTOT for the treatment of sustained low SpO_2_ levels, or “chronic hypoxemia”, with hypoxemia in children younger than one defined as spending 5% of oximetry recording time with SpO_2_ ≤ 93%. In children, the most common indication for LTOT is chronic neonatal lung disease or bronchopulmonary dysplasia (BPD), accounting for 60% [4]. Additional indications in the neonatal period include interstitial lung disease (2% of all children on LTOT) and pulmonary hypertension with or without congenital heart disease (5% of all children on LTOT), although both are based on non-systematic clinical observations. For chronic neonatal lung disease, the provision of LTOT contributes to earlier discharge and reduced health care costs for the 30–40% of very preterm newborns diagnosed with this major preterm morbidity [4,48,49]. In a recent retrospective, whole-population study of all <28 weeks of gestation newborns admitted to an NICU in England between 2014 and 2018, 29.4% of those who survived to discharge went home on LTOT [50]. This is similar to rates of LTOT for this population in Australia and New Zealand [51] and has not changed significantly over the last decade [6]. However, despite the common use of LTOT in this population, its use continues to be based on very low-quality evidence [48].

For near-term or term newborns, data on the use of LTOT is scarce. The largest retrospective cohort study of 48,877 newborns 23–43 weeks’ gestation born in 228 NICUs in the USA found 0.7% (246 of 34,934) near-term or term newborns were discharged on LTOT [6]. While the most recent Australasian data reports a rate of 0.83% in newborns 34–44 weeks’ gestation, newborns must have received assisted ventilation (mechanical ventilation) including intermittent positive pressure ventilation, continuous positive airway pressure, or nasal high flow for four or more consecutive hours to be included in the registry [51]. In this group, clinical risk factors that predicted the likelihood of home oxygen use included need for mechanical ventilation, persistent pulmonary hypertension, and meconium aspiration syndrome [52]. However, it is important to recognise that 93% of near-term and term infants discharged on LTOT never experienced mechanical ventilation [6].

LTOT is also employed in the management of CSA, a condition in which airflow ceases due to lack of respiratory effort, leading to inadequate or absent ventilation and impaired gas exchange. CSA predominantly affects late-preterm and term infants with underlying disorders [53,54], with the reported incidence approximately 1 in 1000 term infants [54]. However, CSA is also diagnosed in infants with no underlying medical condition, where its pathophysiology remains poorly understood [55] and related longer-term outcomes are infrequently reported in the literature [56]. LTOT has been shown to abolish periodic breathing, prevent reactive desaturation, and reduce central apnoea number [57]. In an Australian retrospective cohort of infants < 1 year with no other medical conditions diagnosed with CSA who underwent polysomnography, Hayashi and colleagues reported improved apnoea–hypopnea index, REM and non-REM sleep, and mean oxygen saturations on LTOT [58].

Adequate oxygen supply to optimise outcomes and reduce toxicity is imperative within neonatal medicine. Historically, it has been shown that liberal oxygen use is associated with retinopathy of prematurity and bronchopulmonary dysplasia, whereas insufficient oxygen has been associated with neurodevelopmental impairment and increased mortality. Prolonged oxygen exposure carries the risk of oxygen-related injuries to the infant. Oxidative stress occurs when the production of reactive oxygen species exceeds the capacity of antioxidant defences, leading to cellular damage [59]. In neonates, pulmonary vascular development is sensitive to excessive oxygen levels. The STOP-ROP (supplemental therapeutic oxygen for pre-threshold retinopathy of prematurity) multicentre randomised control trial investigated the effects of higher oxygen saturation targets (96–99%) versus conventional targets (89–94%) in preterm infants with pre-threshold retinopathy of prematurity [60]. The study observed an increased risk of adverse pulmonary events in the higher saturation group, suggesting careful titration is required to ensure normal pulmonary vascular development [60]. The heterogeneity of the neonatal population may influence their physiological response to oxygen, making it difficult to ascertain an infant’s risk to oxygen injury [61]. Although LTOT can facilitate earlier discharge, this treatment can be burdensome on the caregiver. Extensive education is required prior to discharge to ensure effective management of LTOT and appropriate responses to an infant’s oxygen needs [62]. A prospective study found that the demands of complex home care (including LTOT) following NICU discharge were associated with reduced caregiver quality of life [63]. Balancing the need to prevent oxygen toxicity with the practical demands placed on caregiver LTOT management is an important consideration in this context.

Titration or weaning of LTOT is also contentious, with a lack of evidence on which to base treatment guidelines [64]. As a result, approaches differ between hospitals [65], despite the availability of consensus-based recommendations such as those from the Thoracic Society of Australia and New Zealand for infants with chronic neonatal lung disease (Table 3) [31]. While limited evidence supports an SpO_2_ value ≥ 92% 40 min into a trial of air best as the best predictor of readiness for ceasing oxygen in infants with improving BPD [66], approaches remain inconsistent ranging from weaning to night-time-only LTOT versus maintenance of continuous oxygen use until the infants have no requirement for supplemental oxygen at all [4]. Depending on individual cases and clinical practice, infants on LTOT will most likely follow a monitoring plan that includes follow-up overnight oximetry or polysomnography to guide decisions on oxygen need. Despite this variability in clinical practice, most infants begin to wean from supplemental home oxygen by 6 months of age [31,67].

### 6.2. Caffeine Therapy for Near-Term Infants

Methylxanthines, such as caffeine, are commonly used in clinical care to treat preterm infants who experience apnoea of prematurity due to immature respiratory control. Caffeine therapy is a non-invasive, cost-effective, well-tolerated intervention for the prevention and treatment of apnoea of prematurity and IH, with a long pharmacological half-life and low toxicity [68]. While the exact mechanisms of action are complex, at standard doses, caffeine exerts its respiratory stimulant effect by competitively blocking A_1_ and A_2_ adenosine receptors within the brainstem, which play a key role in respiratory control [69]. Critically, in very preterm newborns, caffeine decreases the incidence of chronic lung disease and improves survival free of neurodevelopmental delay including cerebral palsy and cognitive delay [69,70]. However, there are still aspects of caffeine use in the neonatal population that are unresolved, including significant intersubject variability with respect to its effectiveness in reducing the frequency of apnoeic episodes [71], the optimal dose [72], and whether there is a need for therapeutic drug monitoring [73].

While there remains considerable variation in clinical practice in relation to the target population and duration of treatment [74], the predominant patient group receiving caffeine therapy are those preterm newborns < 34 weeks’ gestation. The Caffeine for Apnoea of Prematurity (CAP) trial investigated the impact of caffeine treatment during the acute illness stage following preterm birth, though not the frequency of IH during or immediately after the intervention period, with improved neurodevelopmental outcome and decreased white matter injury [70,75]. However, the Canadian Oxygen Trial (COT) investigating the impact of lower or higher arterial oxygen saturations on the rate of death or disability in extremely preterm infants, found that the association between IH and later neurodevelopmental sequelae was greatest at later PMA when caffeine was more likely to have been discontinued [43,74]. Despite this, the use of caffeine to treat IH in more mature newborns has received limited attention. In a prospective, multicentre randomised controlled trial, Rhein and colleagues investigated the impact of extended caffeine treatment compared to usual timing of caffeine discontinuation in infants born < 32 weeks’ gestation [76]. Prolonging the duration of caffeine therapy was associated with a nearly 50% reduction in the amount of time spent with O_2_ saturations < 90% at 35 and 36 weeks’ PMA [76].

More recently, the recognition that more mature newborns experience frequent episodes of IH until they reach term-corrected age [23] has focused attention on the use of caffeine in older newborns [77]. Oliphant and colleagues investigated the optimal daily caffeine dose to reduce IH in near-term infants (34+0–36+6 weeks’ gestation) [77]. Both 10 and 20 mg/kg/day decreased the mean rate of IH (defined as a fall in SpO_2_ ≥ 10% below baseline) at two weeks in comparison to the placebo group [77]. Further, the 20 mg/kg/day dose increased mean SpO_2_ (97.2 (1.0) vs. placebo 96.0 (0.8); *p* < 0.001) and reduced the percentage of time SpO_2_ < 90% (0.5 (0.2–0.8) vs. 1.1 (0.6–2.4); *p* < 0.001) at two weeks [77]. The beneficial effects on the measures of respiratory instability were higher in the 20 mg/kg/day dosage, suggesting that near-term infants require a higher dose of caffeine than preterm infants, likely due to drug elimination increasing with advancing gestational age [77]. Whether these effects translate into improved neurodevelopmental outcomes remains unknown [23,78].

### 6.3. Is the Current Approach to Diagnosis of IH in the Near-Term to Term Newborn Appropriate?

As outlined above, the current diagnosis and management of IH in the late-preterm and term newborn is impacted by the inherent limitations of pulse oximetry. The lack of established normal reference ranges for overnight oximetry in this patient group, inconsistencies in the definition of what constitutes IH, and uncertainty regarding the degree of IH associated with long-term adverse neurodevelopmental outcome contribute to its limitations. Oxygen saturation is an upstream of metabolism, and misconceptions on the inherent value and interpretation of pulse oximetry may limit an integrated understanding of oxygen delivery, utilisation, and metabolic demand. Coupled with known variation in physician interpretation [79], the current approach to diagnosis of IH and subsequent management in this population likely leaves room for improvement. With the relationship between IH and later adverse neurodevelopmental outcome as the primary driver for investigation and subsequent management, the lack of end-organ specificity of pulse oximetry is also an important consideration. NIRS has the potential to address these limitations. By providing real-time, non-invasive measurements of regional mixed arterial-venous oxygen saturation, NIRS can reveal tissue hypoxia earlier than conventional pulse oximetry [80,81]. By using light in the near-infrared band (700–900 nm), NIRS measures the difference between oxyhaemoglobin and deoxyhaemoglobin in a specific tissue [25,82]. NIRS can assess real-time fluctuations in cerebral tissue oxygen saturation (rSO_2_), by placement of a small sensor on the forehead. In paediatric patients, rSO_2_ decreases 15 to 30 s prior to any change in pulse oximetry in response to hypercyanotic episodes or pauses in mechanical ventilation [29], while a 5% decrease in rSO_2_ has been observed up to 52 s before a comparable decrease is detected by pulse oximetry [29]. Alarmingly, in this study, for a 5% decrease in SpO_2_, the average decrease from baseline rSO_2_ was 16% [29].

However, as NIRS measures a mixed arterial-venous signal, normative data for tissue-specific baseline reference ranges for rSO_2_ is an essential requirement, with values from pulse oximetry unsuitable [25,82,83]. The few studies on neonatal rSO_2_ reference ranges have predominantly focused on rSO_2_ measured during the immediate transition after birth, typically the first 10 min of life [84,85,86], with normative data available for specific subgroups of newborns or at specific time points in the postnatal period. In healthy term infants (>37 weeks’ gestation) studied across the first 10 min after birth, rSO_2_ steadily increases, stabilising at 10 min [84,86]. From 24 h of age, cerebral rSO_2_ then decreases to day 5 of life [87]. Critically, these postnatal changes in cerebral rSO_2_ have been shown to be device-specific, requiring definition of reference values for each NIRS device [88].

Interpreting NIRS data in neonates is complex, as consideration of both physiological and technical factors are required. Haemodynamic context of the newborn is essential for interpreting neonatal NIRS data. In a prospective study, preterm infants with haemodynamically significant patent duct had lower rSO_2_ values (62 ± 9%) than matched controls without a patent duct (72 ± 10%), which increased to control measurements within 12 h of treatment [89]. Red blood cell transfusion in preterm infants increases rSO_2_ values [90], highlighting haemoglobin concentration is an important factor to consider when interpreting NIRS measurements. Furthermore, NIRS is vulnerable to extracerebral contamination as the near-infrared light must pass through the extracranial tissues (scalp, skull, and extracerebral vasculature) to reach the cerebral tissue [91]. Similar to SpO_2_, skin pigmentation has been shown to underestimate rSO_2_ in infants [92], with a case study of four preterm infants indicating that hyperbilirubinemia may compromise the reliability of NIRS monitoring [93], highlighting the need for caution when interpreting readings in these contexts. Additional factors, including skin and skull thickness, may further influence the accuracy and reliability of NIRS monitoring [94]. However, both simulation and clinical studies have suggested that a neonate’s skin and skull thickness may not influence overall NIRS measurements [95,96]. In addition to physiological factors, technical aspects can also contribute to variability in NIRS measurements. Minor variation in sensor placement on the forehead, even as small as 1cm, have been shown to be a critical source of variability in rSO_2_ [97]. However, this finding was in an adult cohort and may not be reflective of sensor position variations in neonates. In a clinical setting, routine handling of infants can introduce movement and sensor artefacts [25], similar to oximetry, and while software may correct for this, the lack of transparency in algorithm design can make cross-study comparisons difficult. Currently, the main barriers to clinical implementation include the lack of standardised reference ranges in the neonatal population. As such, rather than relying solely on absolute values, NIRS is often used to monitor trends from baseline. When interpreted in the context of relevant physiological and technical considerations, NIRS may offer meaningful insights into neonatal cerebral oxygenation.

## 7. Design Considerations for Future Clinical Studies

The available data suggests that rSO_2_ may be superior at detecting alterations in oxygenation in real-time, compared to the current gold-standard of pulse oximetry. Critically, its use is tissue-specific, allowing insight into whether IH detected by pulse oximetry is also associated with intermittent changes in cerebral rSO_2_. Providing insight downstream of metabolism, rSO_2_ critically allows a more comprehensive understanding of oxygen dynamics. Therefore, is there a role for cerebral NIRS as an adjunct measurement in the investigation of near-term and term newborns with IH to better guide clinical intervention?

**Figure 1:**
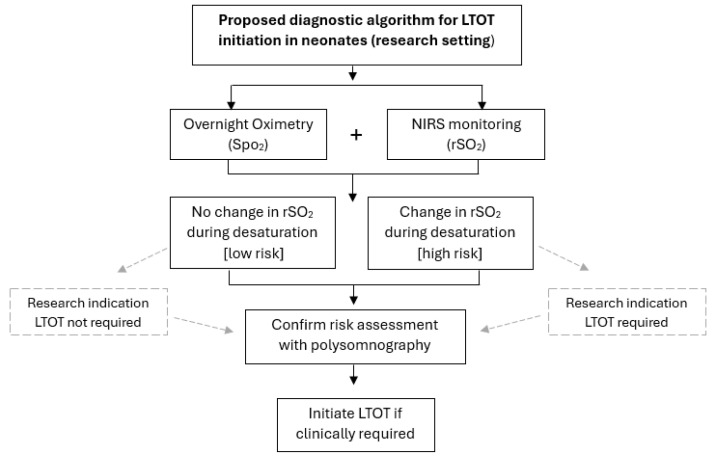
Proposed diagnostic algorithm for LTOT initiation in neonates in a research setting.

With the impact of IH on later neurodevelopmental outcome in the late-preterm and term newborn as the primary driver for investigation and subsequent management, a brain-oriented approach to its diagnosis and management seems logical. Adjunctive cerebral tissue oxygenation may complement pulse oximetry for the purpose of overnight oximetry testing. Pulse oximetry does not reflect regional tissue oxygenation at the cerebral level, and NIRS is known to reliably measure dynamic changes in cerebral tissue oxygen saturation faster than SpO_2_ [98]. Further, there is already evidence for its utility as part of gold-standard polysomnography studies. For example, in paediatric patients undergoing investigation for sleep apnoea, when the commonly used apnoea/hypopnea index was calculated using NIRS-derived rSO_2_, it was greater than that calculated using SpO_2_, suggesting that NIRS does have potential as a valuable adjunct in the assessment of patients with IH [99]. Thus, current diagnostic and treatment approaches to ensure adequate cerebral oxygenation, and by inference normal neurodevelopment, in late-preterm and term newborns with IH may be more accurately achieved through the adjunctive use of NIRS. A proposed diagnostic algorithm for LTOT initiation in neonates in a research setting is listed below in Figure 1. To assess the feasibility of NIRS monitoring to accurately predict an infant’s oxygen needs, we propose adjunctive overnight oximetry and NIRS monitoring. Infants who demonstrate no change in rSO_2_ during desaturation events defined by SpO_2_ would be classified as “low risk”, and LTOT may not be indicated under this hypothesis. Conversely, infants who demonstrate reductions in rSO_2_ during desaturation defined by SpO_2_, may be at higher risk and therefore more likely to require LTOT. Final risk assessment and LTOT requirement would be confirmed by polysomnography.

To improve comparability across studies and enhance clinical utility, adoption of standardised oximetry and NIRS data collection and reporting conventions is essential within neonatal research. This may include clearly stating what device is being used, the averaging time, sampling rate, movement artefacts procedure, illness severity, and haemoglobin concentration. As a result, a better understanding of the relationship between IH and rSO_2_ may be provided by this technology. The implementation of diagnostic scoring systems for rSO_2_, like those used for SpO_2_ overnight oximetry monitoring, may provide useful criteria in investigating IH and the need for LTOT. IH severity can be stratified against scoring systems utilising both rSO_2_ and SpO_2_ values, alongside supporting clinical assessments. By incorporating both oxygen saturation measurements into a comprehensive scoring framework, clinicians can gain a more detailed understanding of the degree of IH and its potential impact on the neonate. This dual approach may offer a more accurate reflection of a newborn’s oxygenation status, thereby improve early detection and guide more targeted interventions. However, baseline and reference ranges are first required to better understand rSO_2_ in the neonatal period. Characterising the response of rSO_2_ to changes in SpO_2_ will enhance our understanding on rSO_2_ as a measure of IH. The influence of the depth and duration of falls in SpO_2_ on rSO_2_ will provide valuable insight into the impact of IH on cerebral oxygen saturation. To determine whether abnormal overnight oximetry accurately reflects episodes of cerebral hypoxia, we propose contemporaneous measurement of SpO_2_ and rSO_2_ during overnight oximetry studies in near-term and term infants with unexplained oxygen requirements. Such an approach will provide better insight into the relevance of SpO_2_-defined IH on cerebral oxygenation and contribute to neonatal reference ranges.

## 8. Conclusions

Within the literature on neonatal IH, the focus has largely been on the effects of oxygen saturation target ranges for treatment. There are challenges with the current mode of diagnostics available, with the relevancy of overnight oximetry being questioned. Currently, no reference ranges exist for this population and the physiological basis for SpO_2_ is limited. With the availability of new technology such as NIRS, the ability to noninvasively measure cerebral oxygenation is accessible. The available literature on neonatal cerebral oxygenation monitoring using NIRS is promising. However, population-based normative data in infants remains absent and our understanding of the relationship between cerebral NIRS and continuous overnight oximetry in newborns with IH is incomplete. The lack of reference ranges creates difficulties in making inferences from cerebral oxygenation measurements in newborns. The use of NIRS as an adjunct to overnight oximetry may provide a more comprehensive downstream assessment of oxygen physiology in neonatal medicine. This knowledge gap could generate potential research to identify the relevancy of current diagnostic methods, which may have downstream effects on treatment options.

## Figures and Tables

**Table 1:** Table of SpO_2_ reference ranges in infants.

Article	Aim	Sample	Methods	Main Findings	Strengths and Limitations
Poets et al. (1996) [1]	Determining oxygen saturation levels for healthy term infants in the first four weeks of life.	Healthy term infants > 37 weeks’ gestation. *n* = 60.	Systemic sampling procedure. Nellcore pulse oximeter (Nellcore Inc., Hayward, CA, USA).	Median baseline SpO_2_, measured during regular breathing was 97.6% (range 92–100) during week 1 versus 98.0% (86.6–100) during week 2–4.	Sample included healthy term infants (2 weeks–24 months). Recording performed in nursey and home environment. Did not collect data on sleep/wake periods. Data limited to device used.
Askie et al. (2018) [33]	To compare the effects of different pulse oximeter oxygen saturation target ranges on death or major morbidity.	Infants born < 28 weeks’ gestation. *n* = 4965.	Prospectively planned individual participant data meta-analysis of five randomised clinical trials (conducted 2005–2014). Masimo pulse oximeter.	Saturation target ranges showed no difference in the primary composite outcome (death or major disability at 18–24 months). Higher targets reduced death and NEC, while lower targets reduced ROP.	Meta-analysis, prospective five trial design. Large sample size. Two trials stopped early. Relatively short follow-up. Device specificity.
Shah et al. (2014) [34]	Determine oxygen saturation profile of healthy late-preterm and term neonates between 12 and 48 h after birth.	Full term (>37 weeks’ gestation) and late-preterm (35–36 weeks’ gestation) infants between 12 and 48 h postnatal age. *n* = 40 term infants. n = 20 late-preterm infants.	Prospective cohort study. 6 h monitoring period using Nellcor OxiMax N-600X pulse oximeter (Covidien, Mansfield, MA, USA) and PROFOX software.	Late-preterm infants spent an average 7% of the time and term infants an average of 4% of the time with SpO_2_ ≤ 90%.	Sample included term infants. Small sample size. Device specificity.
Hunt et al. (2011) [35]	Report longitudinal home recordings of haemoglobin O_2_ saturation by pulse oximetry during sleep in preterm and term infants.	Data from CHIME study. Preterm infants < 1750 g and <35 weeks PMA and healthy term infants. *n* = 103 preterm. *n* = 99 term.	Longitudinal cohort study. Ohmeda Minx pulse oximeter (Ohmeda Corp, Liberty Center, New Jersey, USA).	Median baseline SpO_2_ was approximately 98% for both the preterm and term groups.	Multicentre study (five clinical sites). Only 3 min recordings from each hour. Movement artefact was present in 41% of recordings. Device specificity.
Hunt et al. (1999) [36]	Describe SpO_2_ in the first 25 postnatal weeks and assess the relationships among SpO_2_, breathing pattern, heart rate, and sleep position in healthy term infants.	Healthy term infants enrolled in the CHIME study born at 38–42 weeks’ gestation, ≤30 days at enrolment. *n* = 64 study group. *n* = 150 healthy term infants.	Longitudinal cohort study. Ohmeda Minx pulse oximeter (Ohmeda Corp, Liberty Center, New Jersey, USA).	Median baseline SpO_2_ was 97.9%. Acute decreases in SpO_2_ occurred in 59% of infants, and are correlated with younger age, periodic breathing and apnoea appear to be part of normal breathing and oxygenation behaviour.	Multicentre study (five clinical sites). Term infants included in sample. Only 3 min recordings from each hour. Device specificity.
Evans et al. (2018) [15]	To determine sleeping saturation indices in healthy infants using a modern pulse oximeter with motion artefact extraction technology.	Healthy term infants (>37 weeks’ gestation). *n* = 45.	Prospective cohort study. Nocturnal pulse oximetry at home at 1 month of age and repeated at age 3–4 months. Masimo Rad-8 Oximeter (USA).	Mean oxygen saturation at 1 month is 97.05% (96.59 to 97.52) and 97.65% (97.19 to 98.12) at 3–4 months. Desaturation indices are higher in young infants than older children, and decrease by 3–4 months of age but are still higher than older children.	First to report both 4% and 3% desaturation indices in young infants. Parent-led in the home environment. Sleep/wake interpretation is limited. 4 h minimum recordings. Loss of follow-up. Device specificity.

NEC, Necrotising Enterocolitis; ROP, Retinopathy of Prematurity; CHIME, Collaborative Home Infant Monitoring Evaluation.

**Table 2:** Table of IH and impaired neurodevelopmental outcome in infants.

Article	Aim	Sample	Methods	Main Findings	Strengths and Limitations
Poets et al. (2015) [43]	To determine the association between intermittent hypoxemia or bradycardia and late death or disability.	Infants born 23–27+6 weeks’ gestational age who survived to postmenstrual age of 36 weeks. *n* = 1019.	Post hoc analysis of data from the inception cohort assembled for the Canadian Oxygen Trial. Bayley Scales of Infant and Toddler Development III at corrected 18 months. Masimo pulse oximeter.	Prolonged hypoxemic episodes (at least 1 min) during the first 2 to 3 months after birth were associated with adverse 18-month outcomes (late death or disability).	16 s averaging time. Post hoc design. Socioeconomic variables not considered. Device specificity.
Hunt et al. (2004) [44]	To determine if infants with cardiorespiratory events detected by home memory monitoring during early infancy have decreased neurodevelopmental performance at 1 year of age.	Data from CHIME study. Preterm infants < 1750 g and ≤34 weeks healthy term infants. *n* = 138 term. *n* = 118 preterm.	Home monitoring of cardiorespiratory events. Bayley Scales of Infant Development II at 92 weeks’ post-conceptual age. Ohmeda Minx pulse oximeter(Ohmeda Corp, Liberty Center, New Jersey, USA).	Having 5+ conventional events is associated with lower adjusted mean differences in MDI in term and preterm infants.	Multicentre study (five clinical sites). Interrater reliability tested. Sample includes term infants. The influence of demographic factors on low follow-up rates. Home monitoring limitations. Event thresholds may miss clinically relevant desaturations. Device specificity.
Piteo et al. (2011) [45]	To assess the influence of snoring and sleep duration on developmental outcomes in 6 month old infants.	Infants aged between 0 and 3 months. Non-snoring controls. Snored within the first month of life and continued until 6 months of age. *n* = 88 controls. *n =* 16 snoring infants.	Longitudinal cohort study. Parental-reported snoring. Bayley Scales of Infant and Toddler Development Edition III at 6 months of age.	Snoring during the first 6 months of life was associated with lower cognitive development scores.	Previous protocol lists term infants as sample. Socioeconomic variables analysed. Gestational age at birth not recorded. Parental-reported snoring. Degree of sleep disturbances or desaturations not reported. Small sample of snoring infants.
Montgomery-Downs et al. (2006) [46]	To test the potential association between snoring and developmental performance among infants.	Healthy infants (8.2 ± 0.4 months), born 38.8 (1.5) weeks’ gestational age. *n* = 35.	Longitudinal prospective cohort study. Initial screening survey completed over 6 sites. Bayley Scales of Infant Development II and polysomnography at 8 months of age. Arousals were scored manually defined by the American Sleep Disorders Association Task Force report.	In 8 month old infants, snoring without apnoea or hypopnoea is linked to lower MDI scores when it leads to sleep fragmentation, and is exacerbated by exposure to cigarette smoke.	Gold-standard polysomnography. Correlated against exposure to environmental smoke. Small sample size, limits generalisability. First survey response rate is unknown.
Smith et al. (2014) [47]	To evaluate the relationship between sleep-disordered breathing in early infancy and outcomes at 3 years of age in children with cleft lip and/or palate.	Children with cleft lip and/or palate. Healthy term controls (from a previously published sample). *n* = 33 infants with cleft lip and/or palate. *n* = 156 controls.	Longitudinal prospective cohort study. Diagnostic polysomnography at infancy. Bayley Scales of Infant and Toddler Development III at 3 year follow- up.	Polysomnography in infancy was correlated with outcomes at 3 years of age; lower percentage of AS/REM sleep was associated with lower cognition score; more obstructive events were associated with lower global behaviour ITQOL score; and higher number of respiratory events in infancy was associated with reduced weight z-score.	Extended follow-up (3 years). Results only generalisable to infants with clef lip and/or palate. Possible selection bias. Males overrepresented in follow-up cohort.

CHIME, Collaborative Home Infant Monitoring Evaluation; MDI, Mental Development Index; AS/REM, Active Sleep/Rapid Eye Movement; and ITQOL, Infant Toddler Quality of Life.

**Table 3:** The Thoracic Society of Australia and New Zealand oxygen weaning recommendations.

1	SpO_2_ is to be maintained within age-appropriate targets over time.
2	SpO_2_ should be assessed by overnight oximetry in the days prior to discharge and have the first clinical review in a 4–6-week window.
3	Thereafter, SpO_2_ is recommended to be reassessed on a 4–8 week basis to ensure adequacy of the supplemental oxygen and allow weaning or increase as appropriate.
4	Twenty four hour oxygen therapy is usually recommended. Some older infants may not tolerate daytime supplemental oxygen and compromising to sleep oxygen therapy may be appropriate to maximise the number of hours per day at target SpO_2_.
5	Supplemental oxygen for infants is usually prescribed in steps including 0.5 (1/2), 0.25 (1/4), and 0.125 (1/8) L/min. In some centres, lower flow rates may be available and appropriately utilised.
6	In general, an overnight oximetry study should be performed on the current prescription first. If a previous oximetry on this flow was recent and found to be at or above target, it may be appropriate to go straight to an assessment on a lower prescription.
7	Whilst SpO_2_ targets are the main guide, notes should also be taken of the infant’s overall clinical picture including work of breathing, feeding, growth, and any co-morbid cardiac disease.
8	Oxygen can be discontinued when target saturation is achieved without it.

## Data Availability

No new data were created or analyzed in this study. Data sharing is not applicable to this article.

## Abbreviations

The following abbreviations are used in this manuscript:

IH	Intermittent Hypoxia
SpO_2_	Peripheral Arterial Oxygen Saturation
LTOT	Low-Flow Oxygen Therapy
NIRS	Near-Infrared Spectroscopy
REM	Rapid Eye Movement
EEG	Electroencephalogram
EOG	Electro-Oculograms
EMG	Electromyogram
ECG	Electrocardiogram
CHIME	Collaborative Home Infant Monitoring Evaluation
PMA	Postmenstrual Age
ASA	Australasian Sleep Association
OSA	Obstructive Sleep Apnoea
CSA	Central Sleep Apnoea
BSID	Bayley Scales of Infant and Toddler Development
MDI	Mental Developmental Index
NICU	Neonatal Intensive Care Unit
BPD	Bronchopulmonary Dysplasia
CAP	Caffeine for Apnoea of Prematurity
COT	Canadian Oxygen Trial
rSO_2_	Cerebral Oxygen Tissue Saturation
